# Effect of the Re-Vitrification of Embryos at Different Stages on Embryonic Developmental Potential

**DOI:** 10.3389/fendo.2021.653310

**Published:** 2021-07-14

**Authors:** Jingyu Li, Shun Xiong, Yanhua Zhao, Chong Li, Wei Han, Guoning Huang

**Affiliations:** Chongqing Key Laboratory of Human Embryo Engineering, Chongqing Reproductive and Genetics Institute, Chongqing Health Center for Women and Children, Chongqing, China

**Keywords:** re-vitrification, human embryo, developmental potential, frozen-warmed embryo transfer, mouse embryo

## Abstract

**Background:**

Using re-vitrified human embryos for frozen-warmed embryo transfer (FET) is a valuable option when there are no other cryopreserved embryos to use, however, except for the PGT cases, no published data are available for FET with human embryos that were re-vitrified at different developmental stages.

**Objective:**

To evaluate the effect of re-vitrification of embryos at different stages on embryonic developmental potential.

**Method:**

This study included clinical retrospective and mouse experimental studies. For the retrospective study, a total of 25 FET cycles with re-vitrified day 3 embryos (re-vitrification group 1) and 54 FET cycles with re-vitrified day 5 blastocysts (re-vitrification group 2) between January 2015 and December 2019 were included in this study. The corresponding FET cycles with once-vitrified embryos were identified using propensity score (PS) matching according to the time of embryo transfer. For the mouse experimental study, we divided embryos into 5 groups: fresh (group 1), vitrified at the 8-cell stage (group 2), vitrified at the early blastocyst stage (group 3), vitrified at the 8-cell stage, and re-vitrified at the 8-cell (group 4) or early blastocyst stage (group 5). The fresh embryos was selected as control group. The primary outcome in this study was delivery outcomes.

**Results:**

No significant difference in delivery rate was detected between re-vitrification group 1 (24.00%) and the corresponding control group (28.00%). However, re-vitrification group 2 (46.3%) showed a significant decrease in delivery rate compared with the two corresponding control groups (63.89% and 64.12%) (*P* < 0.05). Our experiment using mouse embryos also confirmed the clinical data, and showed that re-vitrification at the blastocyst stage following the first round of vitrification at the 8-cell stage reduced the delivery rate. In addition, both re-vitrified groups showed a significantly higher expression level of *BAX*. However, only re-vitrification at the blastocyst stage increased the expression level of *CASPASE3.*

**Conclusions:**

Re-vitrification at the 8-cell and blastocyst stages has different effects on embryonic developmental potential, as re-vitrification at blastocyst stage following a previous vitrification at 8-cell stage reduced the delivery rate, while vitrification at the 8-cell stage twice achieved comparable pregnancy outcomes to the once-vitrified group.

## Introduction

Since the first clinical pregnancy from frozen-warmed embryo transfer (FET) was reported in 1983 (1), embryo cryopreservation has become a fundamental procedure in assisted reproductive technology (ART). FET improved the cumulative live birth rate because it allowed multiple embryo transfers (ETs) in a single stimulation cycle (2, 3). In addition, the application of cryopreservation technology minimized the risk of multiple pregnancies and ovarian hyperstimulation syndrome (4).

The two widely used cryopreservation methods are slow freezing and vitrification (5). Compared with the slow freezing method, vitrification is a simple, inexpensive and fast technique (6). This method allows the solidification of cells and the extracellular milieu into a glass-like state, thus preventing the formation of ice crystals and cell damage (7). Several studies have reported that vitrification has higher survival rates, and better clinical outcomes than slow freezing (5, 8). Therefore, many laboratories worldwide have replaced slow freezing with vitrification as the technique of choice for cryopreserving embryos.

In our center, we have vitrified one to three day 3 embryos per Cryotop tip. In order to avoid the risk of multiple pregnancies, up to two embryos have been transferred per cycle in recent years. Furthermore, single embryo transfer might be requested by patients at the day of FET. As a result, there may occasionally be a surplus of surviving embryos available for re-vitrification that can be transferred in the future. In addition, some day 3 embryos were requested to be warmed and cultured to the blastocyst stage, thus leading to the re-vitrification of blastocysts when a transfer cycle was cancelled. Therefore, the re-vitrification method would be a valuable option to increase the cumulative live birth rate while decreasing the risk of multiple pregnancies. To our knowledge, only limited data are available on FET with re-vitrified human embryos, and most are case reports (9–14). However, there are no data about the effect of re-vitrification at different developmental stages on clinical outcomes.

In this study, we aimed to evaluate clinical outcomes of the re-vitrification of human embryos at the 8-cell or blastocyst stage derived from vitrified-warmed day 3 embryos. In addition, we further explored the effect of re-vitrification at different developmental stages on embryonic developmental potential in a mouse model.

## Materials and Methods

### Study Design and Patient Selection

All the FET cycles using twice-vitrified-warmed embryos from January 2015 to December 2019 at our center were retrospectively reviewed. Women receiving pre-implantation genetic testing (PGT) were excluded. Two re-vitrification groups of patients were included according to the day of the second vitrification: (1) embryos vitrified at day 3 and re-vitrified at day 3 (re-vitrification group 1, n=25), and (2) embryos vitrified at day 3 and re-vitrified at day 5 (re-vitrification group 2, n = 54). For re-vitrification group 1, the control group had FET cycles with vitrified-warmed day 3 embryos and was identified *via* propensity score (PS) matching from a cohort of 23620 ET cycles from 2015 to 2019. For the re-vitrification group 2, two control groups with blastocyst transfer cycles were identified according to the day of vitrification: (1) vitrified day 5 blastocysts identified *via* PS matching from a cohort of 653 cases in which all embryos were frozen and undergone second or greater order of FET (control group 1, n=108), and (2) vitrified day 3 embryos that were cultured to day 5 (control group 2, n = 170).

### Fresh Cycles

All patients used long or short protocols for ovarian stimulation. Oocytes were retrieved through the vagina 36 h after human chorionic gonadotropin (hCG) injection and fertilized using conventional *in vitro* fertilization (IVF) or intracytoplasmic sperm injection (ICSI). Normal zygotes with two pronuclei were cultured in G1 medium (Vitrolife, Sweden) at 37°C with 5% O_2_ and 6% CO_2_ in incubators until ET or vitrification on day 3. We vitrified at maximum of four good-quality embryos on day 3 after transfer per patient, and the remaining embryos were placed in extended culture media. For day 3 embryos, we scored embryos on their blastomere shape, blastomere number, and fragmentation rate. An embryo was defined as grade 1 when it had an even blastomere shape and <10% fragmentation, grade 2 when it had uneven blastomeres and 10–25% fragmentation, grade 3 when it had uneven blastomeres and 25–35% fragmentation, and not recommended for transfer or cryopreservation when the fragmentation >35%. Embryos with grade 1-3 were defined as transferrable embryos.

### Human Blastocyst Culture

Remaining or warmed day 3 embryos were cultured in G2 medium (Vitrolife, Sweden) at 37°C with 5% O_2_ and 6% CO_2_ in incubators until day 5. The blastocyst score was determined according to Gardner’s grading system. Blastocysts reaching the expanded or hatching stage and earning a score above grade CC (inner cell mass/trophectoderm) were cryopreserved by vitrification. All blastocysts were shrunk by laser-assisted hatching to ensure that vitrification was effective.

### Vitrification and Warming Procedure

Vitrification was performed using a commercial kit (Kitazato Company, Japan), in accordance with a previous report (15). Firstly, embryos were transferred to equilibration solution for 12-15 min. Then, the embryos were exposed to the vitrification solution for 45-60 s. Finally, embryos were loaded on the tip of Cryotop with a small volume of vitrification solution and immersed in liquid nitrogen immediately.

The warming of embryos was performed with a four-step protocol. Firstly, vitrified embryo on the tip of Cryotop were dipped into 1.0 M sucrose solution (TS), which had been preheated to 37°C for 2 h, and kept there for 1 min. Secondly, embryos were suspended in 0.5 M sucrose solution (DS) for 3 min, and then, in WS1 for 5 min and WS2 for 1 min, respectively. Finally, were transferred to medium for culture.

### Clinical Follow-Up

Serum concentrations of hCG were measured 14 days after ET. Clinical pregnancy was confirmed by the presence of a gestational sac in ultrasonographic examination at week 4. Pregnancy loss within 12 weeks was defined as early miscarriage. Pregnancy after early miscarriage was defined as ongoing pregnancy. The delivery was defined as the number of achieved live births after 28 weeks of gestation.

### Animals

In this study, male and female ICR mice (6 to 8 weeks old) were purchased from Charles River (Beijing, China). All animals used in this study were maintained and handled according to the policies approved by Chongqing Health Center for Women and Children Hospital.

### Mouse Embryo Collection and Culture

**Figure 1:**
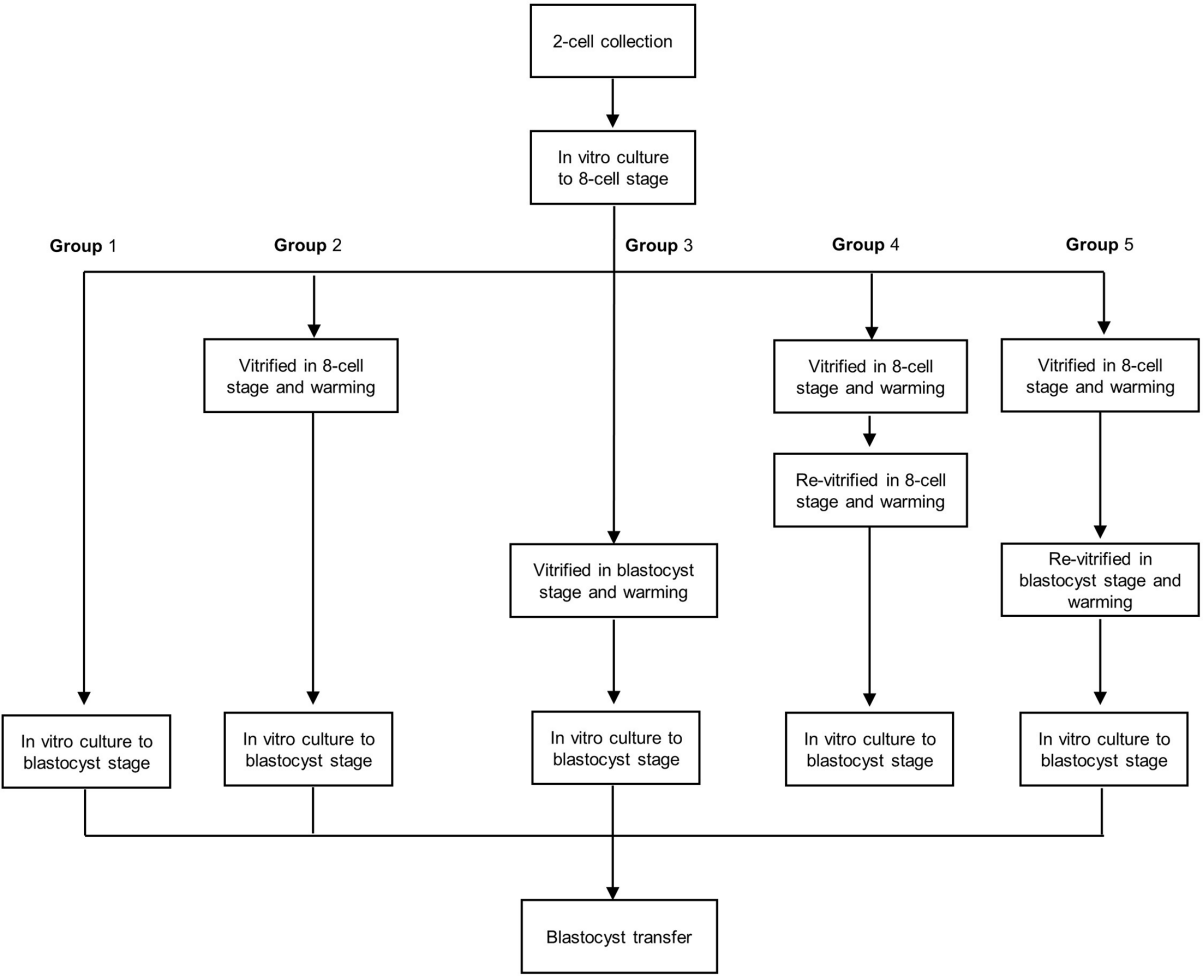
Flow chart of the re-vitrification process at different developmental stages in mouse embryos. Group 1: Fresh embryos; group 2: 8-cell vitrified embryos; group 3: early blastocyst stage vitrified embryos; group 4: vitrified at the 8-cell stage twice; group 5: vitrified at the 8-cell stage and re-vitrified at the early blastocyst stage.

To obtain 2-cell embryos, female mice were superovulated with 10 IU of pregnant mare serum gonadotropin (PMSG; Sigma-Aldrich) followed by 10 IU of hCG (Sigma-Aldrich) 48 h later and mated with fertile males. Pregnancy was evaluated by the presence of a vaginal plug the next morning. Two-cell embryos were recovered by flushing oviducts at 44 h post-hCG and cultured *in vitro* to the 8-cell stage. The 8-cell embryos were divided into to five groups. In group 1, 8-cell embryos were cultured to the blastocyst stage. In group 2, 8-cell embryos were vitrified and warmed and then cultured to the blastocyst stage. In group 3, 8-cell embryos were cultured to early blastocysts, vitrified, warmed, and cultured to the blastocyst stage. In group 4, 8-cell embryos were vitrified and warmed, and live embryos were re-vitrified and then cultured to the blastocyst stage. In group 5, 8-cell embryos were vitrified and warmed, and live embryos were cultured to early blastocysts, re-vitrified, and cultured to the blastocyst stage (**Figure 1**). All embryos were cultured in KSOM medium supplemented with 10% bovine serum albumin (BSA; Millipore, Danvers, MA, USA) at 37°C with 5% O_2_ and 6% CO_2._

### Blastocyst Cell Labeling

An anti-OCT4 antibody and Hoechst 33342 were used to label the inner cell mass (ICM) and total cells of blastocysts. Blastocysts were washed twice in phosphate-buffered saline (PBS), fixed in freshly prepared 4% paraformaldehyde in PBS, permeabilized in 1% Triton X-100 in PBS, and left in blocking solution (1% BSA in PBS) for 1 h. For immunolabelling, blastocysts were incubated overnight at 4°C with an anti-OCT4 antibody (Sc5279, Santa), washed three times, and incubated for 1 h with a secondary antibody, FITC-labelled donkey anti-mouse IgG (A21202, Invitrogen) diluted 1:1000 in blocking solution. Blastocysts were washed and counterstained with 5 g/mL Hoechst 33342. Finally, embryos were mounted on a glass slide and examined using a laser-scanning confocal microscope (Leica TCS SP8).

### Quantitative Real-Time PCR

Total RNA was extracted from oocytes, using the Arcturus PicoPure RNA isolation Kit, according to manufacturer’s instructions (Thermo Fisher Scientific, Waltham, MA, USA), followed by reverse transcription and qRT-PCR, using the PrimeScript RT Master Mix (Takara, Dalian, China) and the SYBR Green qRT-PCR master mix (Takara, Dalian, China) respectively. The amplification cycles were as follows: 95°C for 3 min followed by 40 cycles of 95°C for 15 s, 60°C for 30 s, and 72°C for 20 s, and a final extension at 72°C for 5 min. Relative gene expression was normalized to internal Hprt mRNA levels.

### Mouse Blastocyst Transfer

Blastocysts were transferred into uteruses of day 4 pseudopregnant mice. A total of six blastocysts were transferred to each uterine horn of pseudopregnant mice. Embryos of group 2 were transferred into left horns, embryos of other groups were transferred into the right horn. Surrogate mice were sacrificed on day 18 post-coitus, and rates of implantation and surviving fetuses were calculated.

### Statistical Analysis

The PS matched control database was derived from all FET cycles from January 2015 to December 2019 at our center. PS matching was performed based on female age at ovum pick up (OPU), the number of OPU cycles, endometrial thickness and number of transferred embryos. Control cases were matched to corresponding subjects in the re-vitrification groups based on the closeness of their corresponding propensity scores. The goal was to obtain a 2:1 ratio of control groups to re-vitrification groups.

Continuous variables are presented as the mean ± standard deviation. Categorical variables are presented as *n* (%). For comparisons between the groups, the χ^2^ exact test was used for dichotomous variables, and Student’s t test was used for continuous variables. A P-value < 0.05 was considered significant. All statistical tests were performed using SAS software version 9.3 (SAS Institute, Cary, NC, USA) and SPSS software version 22, 2013 (SPSS, Chicago, IL, USA).

## Results

### Effect of Re-Vitrification at 8-Cell Stage on Clinical Outcomes

There was no difference in the age at OPU, number of OPU cycles performed, endometrial thickness, number of transferred embryos, survival rate, implantation rate, clinical pregnancy rate, miscarriage rate or delivery rate between the re-vitrification group 1 and the control group (**Table 1**).

**Table 1:** Clinical parameters and outcomes of day 3 embryo transfers with once- or twice-vitrified-warmed embryos.

Group	Re-vitrification group 1	Control group (PSM)	*P*-value
No. of cycles	25	50	
OPU age (years)	34.36 ± 6.92	34.34 ± 6.80	NS
OPU cycle order	1.68 ± 1.41	1.72 ± 1.37	NS
Endometrial thickness (mm)	9.12 ± 1.61	9.22 ± 1.57	NS
No. of embryos warmed	48	94	
No. of surviving embryos (%)	48/48 (100%)	92/94 (97.87%)	NS
No. of transferred embryos per ET	1.84 ± 0.61	1.74 ± 0.56	NS
Implantation rate (%)	9/46 (19.57%)	20/87 (22.99%)	NS
Clinical pregnancy rate (%)	7/25 (28.00%)	16/50 (32.00%)	NS
Singletons	5	12	
Twins	2	4	
Delivery rate (%)	6/25 (24.00%)	14/50 (28.00%)	NS
Singletons	4	10	
Twins	2	4	
Miscarriage rate (%)	1/10 (10.00%)	2/16 (12.50%)	NS

Categorical variables are presented as proportion (%). Continuous variables are presented as mean ± SD.

For comparisons of dichotomous variables, χ^2^ test was used. For comparisons of continuous variables, Student’s t test was used.

OPU, ovum pick up; ET, embryo transfer; NS, not statistically significant.

### Effect of Re-Vitrification at Blastocyst Stage on Clinical Outcomes

In the previous frozen-warmed cycle of re-vitrification group 2, a total of 131 vitrified day 3 embryos were warmed, among which 87 embryos developed to the blastocysts meeting the criteria for vitrification (blastocysts formation rate of 66.41%). Delivery rates were significantly lower in the re-vitrification group 2 (46.30%) than in control group 1 (63.89%, P = 0.037) and control group 2 (64.12%, P = 0.030). The clinical pregnancy rate of the re-vitrification group 2 (53.70%) was significantly lower than that of control group 2 (72.94%, P = 0.013), and lower than that of control group 1 (70.37%, P = 0.055) without reaching statistical significance (**Table 2**). The implantation rate of the re-vitrification group 2 (45.59%) was significantly lower than that of control group 1 (63.70%, P = 0.020), and slightly lower than that of control group 2 (58.10, P=0.080) without reaching statistical significance. The miscarriage rate was slightly higher in the re-vitrification group than in control group 1 and group 2, but the differences were not statistically significant (**Table 2**).

**Table 2:** Clinical parameters and outcomes of blastocyst transfers with once- or twice-vitrified-warmed embryos.

Group	Re-vitrification group 2	Control group 1 (PSM)	*P_1_*-value	Control group 2	*P_2_*-value
No. of cycles	54	108		170	
OPU age (years)	30.94 ± 3.80	30.10 ± 3.93	NS	30.76 ± 4.06	NS
OPU cycle order	1.24 ± 0.66	1.18 ± 0.49	NS	1.28 ± 0.67	NS
FET order	2.57 ± 0.91	2.25 ± 0.45	NS	2.08 ± 0.75	NS
Endometrial thickness (mm)	8.87 ± 1.26	8.84 ± 1.23	NS	8.86 ± 1.46	NS
No. of embryos warmed	70	141		908	
No. of surviving embryos (%)	69/70 (98.57%)	139/141 (99.28%)	NS	898/908 (98.90%)	NS
No. of transferred embryos per ET	1.26 ± 0.44	1.26 ± 0.43	NS	1.86 ± 0.34	NS
Implantation rate (%)	31/68 (45.59%)	86/135 (63.70%)	0.020	183/315 (58.10%)	0.080
Clinical pregnancy rate (%)	29/54 (53.70%)	76/108 (70.37%)	0.055	124/170 (72.94%)	0.013
Singletons	27	66		65	
Twins	2	10		59	
Delivery rate (%)	25/54 (46.30%)	70/108 (63.89%)	0.037	109/170 (64.12%)	0.030
Singletons	24	53		65	
Twins	1	7		44	
Miscarriages rate (%)	4/29 (13.80%)	6/76 (7.89%)	NS	15/170 (8.82%)	NS

Categorical variables are presented as proportion (%). Continuous variables are presented as mean ± SD.

For comparisons of dichotomous variables, χ^2^ test was used. For comparisons of continuous variables, Student’s t test was used.

P_1_-value: re-vitrification group vs. control group 1, P_2_: re-vitrification group vs. control group 2.

OPU, ovum pick up; ET, embryo transfer; NS, not statistically significant.

### Developmental Potential of Mouse Embryos After Re-Vitrification

**Figure 2:**
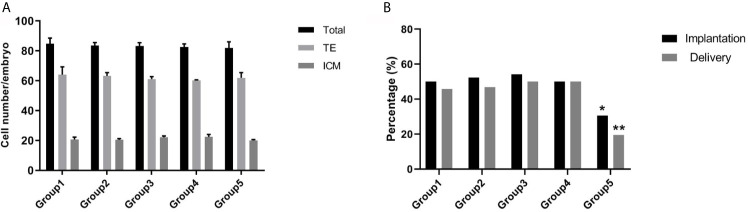
Effect of re-vitrification at different stages on the mouse embryonic development. **(A)** Blastomere numbers were not significantly different among the groups. Values are shown as the mean ± the standard error of the mean (SEM). **(B)** Group 5 showed a significant lower of implantation and delivery rates. Significant difference, *P < 0.05, **P < 0.01.

To further investigate the effect of re-vitrification on embryonic developmental potential, we performed an experimental study using mouse embryos. Re-vitrification and vitrification did not show a significant reduction in expanded blastocyst formation compared to the fresh group. However, the blastocyst hatching rate in group 5 was significantly lower than that in the fresh group (**Table 3**). Furthermore, total cell numbers and ICM percentages were similar among the five groups (**Figure 2A**). There were no significant differences in the implantation and fetuses rates from group 1, group 3, and group 4 compared to group 2. Interestingly, the implantation and delivery rates significantly decreased in group 5 compared to group 2 (30.56% *vs.* 52.38%, P=0.034; 19.44% *vs.* 46.83%, P=0.006; **Figure 2B**), which was similar to our previous observation that re-vitrification at the blastocyst stage following the first vitrification at the 8-cell stage worsened the clinical outcomes.

**Table 3:** Effect of re-vitrification in different stages on mouse embryonic development.

Group	Fresh embryos (n)	No. of surviving embryos (%)	No. of re-vitrified embryos	No. of surviving re-vitrified embryos (%)	No. of expanded blastocysts (%)	No. of hatching blastocysts (%)
1	100	–	–	–	100 (100.00)	98 (98.00%)
2	96	94 (97.92%)	–	–	93 (98.94%)	90 (96.77%)
3	111	108 (97.30%)	–	–	108 (100.00)	99 (91.67%)
4	102	102 (100.00%)	102	102 (100.00%)	100 (98.04%)	98 (98.00%)
5	104	104 (100.00%)	104	100 (96.15%)	96 (96.00%)	86 (89.58%)^*^

Group 1: Fresh embryos; Group 2; 8-cell vitrified embryos; Group 3; Blastocyst vitrified embryos; Group 4; Vitrified at the 8-cell stage and re-vitrified at 8-cell stage; Group 5; Vitrified at the 8-cell stage and re-vitrified at the blastocyst stage. ^*^Significant difference with group 1 (P < 0.05).

### Expression Levels of Apoptotic Genes

**Figure 3:**
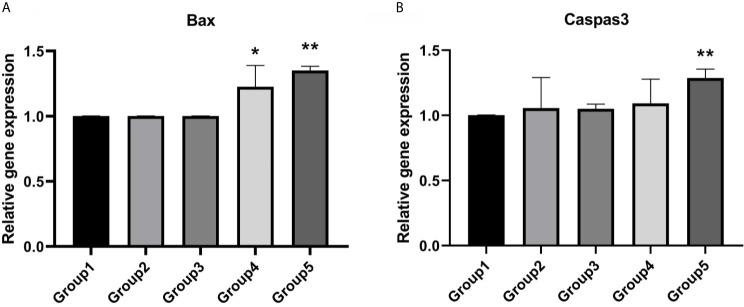
Expression levels of apoptotic genes. **(A)** Group 4 and group 5 showed a significantly higher expression level of *Bax*. Values are shown as the mean ± the standard error of the mean (SEM). **(B)** Group 5 showed a significant higher expression level of *Caspase3*. Significant difference, *P < 0.05, **P < 0.01.

Expression of *BAX* was significantly higher in group 4 (P <0.05) and group 5 (P <0.01) compared to fresh embryos (**Figure 3A**). However, the expression level of *Caspas3* was similar between group 4 and group 1. Only group 5 displayed a significantly higher level of *CASPASE3* compared to group 1 (**Figure 3B**).

## Discussion

With the wider application of cryopreservation technology in ART, the number of cryopreserved embryos with vitrification has rapidly increased. It is sometimes necessary for embryos to be vitrified and warmed twice before transfer. However, little is known about the effect of re-vitrification on the developmental potential of human embryos. In the present study, the results showed that clinical outcomes of the twice-vitrified day 3 embryos did not differ from those of once-vitrified human day 3 embryos. However, delivery rate of vitrified blastocysts, which were vitrified at day 3 and re-vitrified at the blastocyst stage, were significantly lower than those of the once-vitrified control groups.

In clinical research, it is impossible to explore the effect of re-vitrification on *in vitro* embryonic development. Therefore, we performed an experimental study using mouse embryos. We re-vitrified mouse embryos at the 8-cell or blastocyst stage, which corresponded to the two developmental stages of re-vitrification in human embryos. The results showed that only embryos re-vitrified at the blastocyst stage showed a significantly lower blastocyst hatching rate than the fresh group, which may explain the reduction in clinical outcomes after re-vitrification at the blastocyst stage. Moreover, we also observed that re-vitrification at the mouse blastocyst stage resulted in significantly lower implantation and delivery rates, which confirmed our previous clinical results. These results showed that re-vitrification at the blastocyst stage had a notable negative effect on embryonic developmental potential, while re-vitrification at the 8-cell stage did not.

There has been a longstanding debate regarding the effect of re-vitrification on human embryonic development, and clinical outcomes. Zheng et al. (14) compared the clinical outcome of re-vitrified blastocysts derived from frozen-warmed day 3 embryos and fresh embryos, and showed that the live birth rate in the twice-cryopreserved group was significantly lower than that in the control group, which is in line with our results. Murakami et al. (12) also found that cryopreservation twice increased the miscarriage rate compared to cryopreservation once. However, another study reported that implantation and clinical pregnancy rates were comparable between both the re-cryopreserved group and control group (10). It should be noted that in the studies by Zheng et al. (14) and (12) Murakami et al., two cryopreservation methods were used sequentially, including slow freezing and then vitrification. In contrast, Kumasako et al. (10) used two times of vitrification. The different cryopreservation methods may be the possible explanation for the discrepancies between the results. Therefore, more data regarding the effects of multiple vitrification-warming is still needed. In addition, different developmental stages of embryos during cryopreservation may be another possible reason for the different results. Similar to our study, Zheng et al. (14) and (12) Murakami et al. frozen the embryos at the cleavage stage and re-frozen at the blastocyst stage. However, Kumasako et al. (10) vitrified 2 pronuclear (2PN) stage zygotes and re-vitrified them at the blastocyst stage. We hypothesized that the 2PN frozen might offer a longer time for recovering the cryoinjury in the process of *in vitro* culturing, compared with the frozen at cleavage stage.

The developmental stage is believed to play an important role in successful vitrification and the subsequent development after warming (16). We re-vitrified embryos at the 8-cell and blastocyst stages, and assayed the effect of the embryonic stage during the re-vitrification process on the embryo developmental rate. Our findings showed that in both human and mouse embryos, only embryos vitrified at 8-cell stage and re-vitrified at the blastocyst stage displayed reduced implantation and delivery rates, which suggested that freezing damage was cumulative. Several studies have demonstrated that vitrification can increase the incidence of aneuploidy (17, 18). Thus, to evaluate the effect of re-vitrification on the embryos, especially for aneuploidy, we excluded the patients receiving PGT in this study. Interestingly, study by Wilding M et al. (19) found that the embryos re-vitrified at the blastocyst stage, which were euploidy confirmed by PGT-A, achieved a comparable pregnancy outcomes to the normal PTG-A group. Combined with our results, we reasoned that the decreasing of clinical outcomes in re-vitrification group 2, might be relevant for the effect of re-vitrification on aneuploidy. Therefore, filtering the aneuploidy blastocysts through PGT-A can increase the clinical outcomes, as reported by Wilding M et al. (19). Except for the aneuploidy, the transcriptome also be affected by vitrification. Many studies have demonstrated that vitrification has negative impact on the expression of genes regulating apoptosis (*P53*, *BCL2L1*, *BAX* and *BCL2*) (20–22), zygotic genome activation (*EIF41AX* and *FIGLA*) (20), pluripotency (*OCT4, SOX2* and *NANOG*) (20, 21), cell differentiation (*KRT19*, *CLDN23*) (23), and implantation (*PTGS2*, *CALB1*) (17, 23), which were essential for the embryonic development. In our study, we also find the significantly higher expression levels of *BAX* in two re-vitrified groups, which in line with previous publications (24, 25). Interestingly, our results showed that only the mouse embryos re-vitrified at the blastocyst stage showed a significantly higher expression level of Caspase3 than the fresh group, which might has a relation to the decreased developmental potential of embryos re-vitrified at the blastocyst stage. During the process of compaction, the morula can implement mechanisms of self-correction to reduce aneuploidy (26). Therefore, we postulated that the damage derived from the first round of cryopreservation might be self-corrected during subsequent development, especially in the morula stage; thus, re-vitrification will reduce the embryonic developmental potential when it occurs after the morula stage.

There were two limitations of this study. First, this was a retrospective study. Therefore, we performed PS matching to minimize selection bias. Second, the re-vitrification group contained patients who had used all of their once-vitrified embryos and had many implantation failures.

In summary, our study showed that re-vitrification at the 8-cell and blastocyst stages has different effects on embryonic developmental potential, as re-vitrification at blastocyst stage reduced the pregnancy rate, while re-vitrification at the 8-cell stage achieved comparable pregnancy outcomes to the once-vitrified group. The mouse experiment also confirmed these clinical results. Therefore, we stress the need to avoid the re-vitrification of blastocysts after a previous vitrification at 8-cell stage when possible. Long-term follow-up studies with more participants are needed to confirm these results and the safety of the re-vitrification procedure.

## Data Availability Statement

The raw data supporting the conclusions of this article will be made available by the authors, without undue reservation.

## Ethics Statement

The studies involving human participants were reviewed and approved by Institutional Review Board (IRB) of Chongqing Health Center for Women and Children Hospital. The patients/participants provided their written informed consent to participate in this study. The animal study was reviewed and approved by Ethics Committee of Chongqing Health Center for Women and Children Hospital. Written informed consent was obtained from the individual(s) for the publication of any potentially identifiable images or data included in this article.

## Author Contributions

JL conceived, designed the study, and performed the data statistics. SX and WH collected and cleaned the clinical data. YZ and CL performed mouse experiment. JL and GH contributed to manuscript drafting with the help from all authors. All authors contributed to the article and approved the submitted version.

## Funding

This study was supported by the Chongqing Science and Health Joint Project (2021MSXM072) and Special Research Project of Chongqing Health Center for Women and Children (2019YJMS01). No competing interests declared.

## Conflict of Interest

The authors declare that the research was conducted in the absence of any commercial or financial relationships that could be construed as a potential conflict of interest.
